# Exploring Neurofeedback Training for BMI Power Augmentation of Upper Limbs: A Pilot Study

**DOI:** 10.3390/e23040443

**Published:** 2021-04-09

**Authors:** Hongbo Liang, Shota Maedono, Yingxin Yu, Chang Liu, Naoya Ueda, Peirang Li, Chi Zhu

**Affiliations:** 1 Maebashi Institute of Technology, Center for Regional Collaboration, 460-1 Kamisadori, Maebashi, Gunma 371-0816, Japan; 2 Department of Systems Life Engineering, Graduate School of Engineering, Maebashi Institute of Technology, 460-1 Kamisadori, Maebashi, Gunma 371-0816, Japan; m1856012@maebashi-it.ac.jp (S.M.); m1956002@maebashi-it.ac.jp (Y.Y.); 3 Department of Environment and Life Engineering, Graduate School of Engineering, Maebashi Institute of Technology, 460-1 Kamisadori, Maebashi, Gunma 371-0816, Japan; m1756503@maebashi-it.ac.jp (C.L.); m1956502@maebashi-it.ac.jp (N.U.); m2056504@maebashi-it.ac.jp (P.L.); 4 Department of Systems Life Engineering, Maebashi Institute of Technology, 460-1 Kamisadori, Maebashi, Gunma 371-0816, Japan

**Keywords:** BMI, EEG, neurofeedback, power augmentation, training, upper limbs

## Abstract

Electroencephalography neurofeedback (EEG-NFB) training can induce changes in the power of targeted EEG bands. The objective of this study is to enhance and evaluate the specific changes of EEG power spectral density that the brain-machine interface (BMI) users can reliably generate for power augmentation through EEG-NFB training. First, we constructed an EEG-NFB training system for power augmentation. Then, three subjects were assigned to three NFB training stages, based on a 6-day consecutive training session as one stage. The subjects received real-time feedback from their EEG signals by a robotic arm while conducting flexion and extension movement with their elbow and shoulder joints, respectively. EEG signals were compared with each NFB training stage. The training results showed that EEG beta (12–40 Hz) power increased after the NFB training for both the elbow and the shoulder joints’ movements. EEG beta power showed sustained improvements during the 3-stage training, which revealed that even the short-term training could improve EEG signals significantly. Moreover, the training effect of the shoulder joints was more obvious than that of the elbow joints. These results suggest that NFB training can improve EEG signals and clarify the specific EEG changes during the movement. Our results may even provide insights into how the neural effects of NFB can be better applied to the BMI power augmentation system and improve the performance of healthy individuals.

## 1. Introduction

An ideally practical Brain-Machine Interface (BMI) should be non-invasive, reliable, convenient, easy to use, and compatible with users. The brain consists of about 100 billion nerve cells, which are called neurons. Signals are generated when the neurons are excited. With the advancement of sensor technology, now the voltage fluctuations generated by the activation of neurons can be measured on the scalp, and these potential signals are called electroencephalography (EEG) [1]. Thus, EEG signals include rich information about the electrical activities of the brain. Since the EEG-based BMI is relatively cheap, safe, and has a good time resolution, so far, EEG-based BMI has been mainly used for functional recovery and mental health improvement, such as the training for post-stroke rehabilitation [2], hearing aids for deaf people [3], and so on. Besides the applications for treatment and recovery, the assistive technology using EEG-based BMI for disabled people has a greater potential for “human enhancement” of healthy people. For example, when transferring a patient from a bed to a wheelchair in caregiving or when carrying heavy objects in logistic centers or construction sites, our upper limbs endure a large amount of strain. In such cases, power augmentation (PA) is of importance. Though it is believed that power augmentation will play a substantial role in human’s daily activities, the number of available BMI applications targeting the power augmentation for healthy people is few so far. Until now, the EEG features and the methods for the BMI PA decoder haven’t been investigated extensively. We tried to extract the EEG features, and then successfully estimated electromyography (EMG) signals from EEG signals of the elbow joint [4], and the shoulder joint [5,6], respectively. With these estimated EMG signals, the BMI PA with an EMG-controlled PA device is expected to be realized.

However, in the application promotion, the results showed the control accuracy and the response speed of the BMI system varied greatly, especially for the subjects without BMI experience. In BMI PA system, the power augmentation device isn’t a simple replacement for the physical limbs. It is a new integrated human-machine system formed by the upper limbs and the power augmentation devices. Thus, even with the best BMI decoder, the BMI PA depends significantly on the subjects’ ability to voluntarily modulate their EEG signals. Because background EEG activity and other sources of electronic noise often fluctuate unpredictably, subjects need to generate EEG signals that are easy to detect to control PA devices. This leads to needing subjects to practice and train repeatedly. On the other hand, the human is good at understanding training goals and the cognitive ability will also continue to improve when a task is continuously processed within a certain period [7]. Therefore, rather than simply collecting EEG data to train the BMI decoder, it is better to train the BMI users directly. From this point of view, it is important to explore how to design and perform BMI training for power augmentation.

Human can control their movements well because the human’s brain receives feedback through the sensory nervous system [8]. Thus, it would be desired for BMI training to get feedback for power augmentation. BMI operation is a neurofeedback (NFB) application. NFB converts neural signals into visual, auditory, and tactile information, and the subject can selectively enhance or inhibit certain components to promote learning and regulate brain function through real-time NFB training (NFBT) [9]. EEG-NFB is the only method widely used in clinical practice because of its portability, high time resolution, and real-time recording and feedback. It is known that NFB training will change the structure and working of the brain, and the generation of new synaptic connections or the improvement of the efficiency of original synaptic connections will lead to permanent changes in neural activity, which is the mechanism of neural plasticity [10]. The signals from a large number of neuronal activities can remain relatively stable for a relatively long time [11]. Especially, because of its endogenous, the effect may be consolidated over time [12]. Therefore, EEG-NFB training is helpful to generate stable specific EEG changes effectively, which can be used as the feedback for BMI training.

Moreover, because almost 50% of the brain is involved in visual processing, and 70% of all the sensory receptors are in the eyes [13], in which the sense of a visual scene can get in less than 0.1 s [14], vision is considered as the most important and effective way for humans to obtain information and closely related to the neuron network compared with hearing, touch, and others. Therefore, vision-based EEG-NFB is considered as the most common and effective feedback training method [15,16], which will be used in our study.

Although “optimal” training setting data for BMI PA do not exist, the training process can be designed and adapted by understanding the NFB underlying principles. Absolutely, clear and stable EEG features are desired and should be used for NFB training. However, the EEG features for power augmentation of the elbow and the shoulder joints were still unclear. Many studies have been carried out to try to find the motion-related EEG features using a variety of methods, including Fast Fourier transform (FFT) [17], Short-time Fourier transform (STFT) [18], wavelet transform [19], Independent Component Analysis (ICA) [20], auto-regressive method [21], Principal Component Analysis (PCA) [22], time-frequency Distributions (TFD) [23], eigenvector methods [24], and so on. To obtain accurate classification results without losing important information at a reasonable time, the speed and accuracy of the feature extraction stage are crucial. FFT is not good at examining non-stationary signals. Therefore, STFT is introduced as a solution for unstable signals. Because STFT basically extracts a frame of the original signals and analyzes it using a window that shifts with time. If the used time window is narrow enough, each frame of the data can be evaluated as stationary, so that the Fourier transform can be used [25]. Moreover, wavelet transform is also a solution for unstable signals, since it can ensure proper time-frequency resolution in all frequency ranges. But this method needs to choose an appropriate mother wavelet, and the Heisenberg Uncertainty reduces its performance. ICA is reasonable for EEG signal analysis because EEG signals measured from multiple electrodes at the scalp are linear sums of temporally independent components. These components are generated by spatially fixed or overlapping brain areas, and the propagation time delay is negligible. Moreover, because the artifacts and EEG signals have completely different generating mechanisms, they are independent. These reasons help in identifying the independent EEG source signals and also noise separation from EEG signals. Consequently, ICA is well suited for feature extraction when it gives better stability and reliability. The auto-regressive method suffers from speed and is not always suitable for a real-time system. On the other hand, PCA is a useful method for improving signal similarity and reducing dimensions of data without the loss of important information. Even in some ICA algorithms, PCA is used for signal preprocessing. But the meaning of each principal component is vague, not as explanatory as the original data. Non-principal components with small variances may also contain important information of the differences, because dimension reduction may have an impact on subsequent data processing. The TFD can analyze relatively long continuous EEG signals when the signal dynamics change rapidly. However, a good resolution is required both in time and frequency, which makes it not applicable in many cases. The eigenvector methods can produce a high-resolution spectrum even when the signal-to-noise ratio (SNR) is low. However, this method may produce spurious zeros, resulting in poor statistical accuracy. Thus, each method has its specific advantages and disadvantages, making it appropriate for specific signals. Hence, it is difficult to determine the priority of methods according to the capability of the method. Moreover, the performance is also due to the specific task. When discussing the performance of a feature extraction method, it is crucial to be clear about the signals to be analyzed. Therefore, the optimum method for any application may be different. Because the EEG features for the power augmentation are still unclear so far, one of the goals of this study is to clarify the distinguishable changes in the EEG signals related to the motion. As EEG signals are non-stationary [26], the most suitable way for feature extraction is to use the time-frequency domain methods. Time-frequency analysis can observe and confirm the EEG changes both in the time and frequency domain at the same time. The results of this study will chart the way forward on features extraction in the next step.

Many studies have shown that perception, cognition, motion, and emotional processing are all related to the specific oscillation patterns, which can be observed in the nervous system. One of the most studied brain patterns to date is alpha activity (8–12 Hz) [27]. Open eyes, increased visual stimulation, and attention, will weaken the alpha activity at parietooccipital [28]. This conclusion also responds to motor tasks [29] and different cognitive needs (such as attention and memory tasks) [27]. Similarly, the relevance between the beta waves and the motion is also examined [30]. Another reason to use sensorimotor rhythm (SMR) (13–15 Hz) is that it is typically modulated by both overt and covert motor activity (that is, both actual and imagined movements), but unaffected by changes in visual stimulation [31]. This means that people can use a BMI while watching a movie, focusing on a friend, browsing the web, or doing other visual tasks. Moreover, there is a greater effect on SMR by watching a realistic moving hand compared with the observation of abstract feedback in the form of a moving bar [32]. Therefore, during the training, we did not use the abstract moving bar on a display or a screen, but use a realistic robotic arm for feedback. Furthermore, it is found that the training group that uses NFB training based on the alpha waves, beta waves, and theta waves performs better in the National Aeronautics and Space Administration (NASA) job tests and has a lower cognitive burden [33]. The results of our previous study [5] also showed that there are motion-related components in 0.1–40 Hz of EEG signals during the motion. Therefore, in this study, the frequency band (0–40 Hz) was used for NFB at first to find out the specific EEG changes of the elbow and the shoulder joints which have not been investigated yet, and then these changes will be used for NFB training and the verification of the training effect.

The specific target application of this study is to control a power augmentation device of upper limbs to follow the human’s movement in healthy people by EEG signals. Thus, the goal of this pilot study was to clarify and evaluate the specific EEG changes when both arms are performing carrying a heavy object with power augmentation simultaneously through NFB training. Therefore, in this paper, first, we describe how to construct a BMI PA system with EEG-NFB. Then we use NFB and BMI to clarify the specific EEG changes by NFB training and evaluate the training effects. Finally, we discuss the influence of this NFB effect on the design of BMI decoder and BMI training.

## 2. Materials and Methods

### 2.1. Participants

Six healthy young males (without BMI experience) were recruited for this study. All subjects were informed of the experimental protocols and consented to the study. This research was also approved by the Institutional Review Board at the Maebashi Institute of Technology. All subjects in this study are the first time to use the BMI system. Subjects were randomly divided into 2 experimental groups, a trainee group and a control group (non-trainee), which have 3 subjects in each group (age, trainee group: 25.0 ± 1 years old, non-trainee group: 27.0 ± 7 years old; body weights, trainee group: 71.3 ± 4.16 kg, non-trainee group: 71.7 ± 3.51 kg; heights, trainee group: 175 ± 5.00 cm, non-trainee group: 174 ± 4.93 cm). The control group did not have NFB training.

### 2.2. System Construction

**Figure 1:**
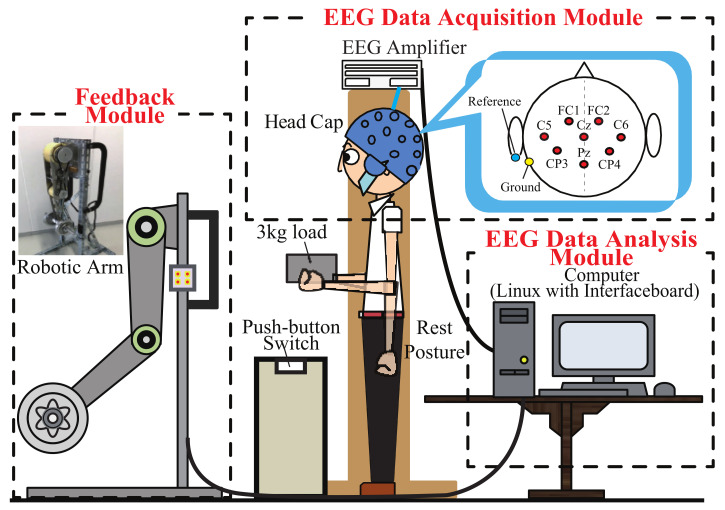
Overview of the constructed system “NFB-BMI-PA 2020”.

The constructed system is shown in Figure 1, which is named “NFB-BMI-PA 2020”. It consists of the following three modules: EEG data acquisition module, EEG data analysis module, and feedback module. Real-time data synchronization, high-speed EEG data acquisition/transmission, and the feedback control of the robotic arm were realized through a multiple-functional interface-board (HRP Interface Board 07-0003-1) of ZUCO (Zuko Co., Ltd., Isehara, Japan) on a Linux running computer. The Linux combined with real-time application interface (RTAI) can ensure the system continuously record and analyze the EEG signals to perform NFB in real-time.

#### 2.2.1. System Software Architecture

**Figure 2:**
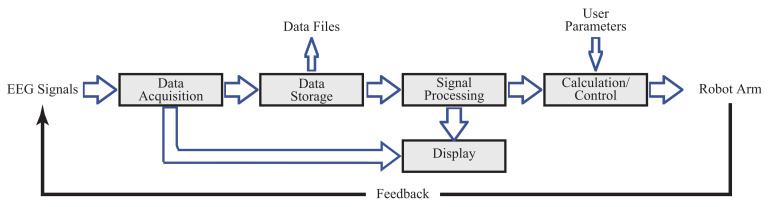
The software architecture of “NFB-BMI-PA 2020”.

We built up a software platform based on the centOS of the Linux environment. This platform was used to process the acquired EEG signals and convert them into robotic arm motion in real-time for feedback. The software architecture is shown in Figure 2. which included EEG data acquisition, data storage, signal processing, calculation/control, and display. The acquired signals were also saved as data files for off-line data analysis.

#### 2.2.2. Real-Time EEG Data Acquisition

**Figure 3:**
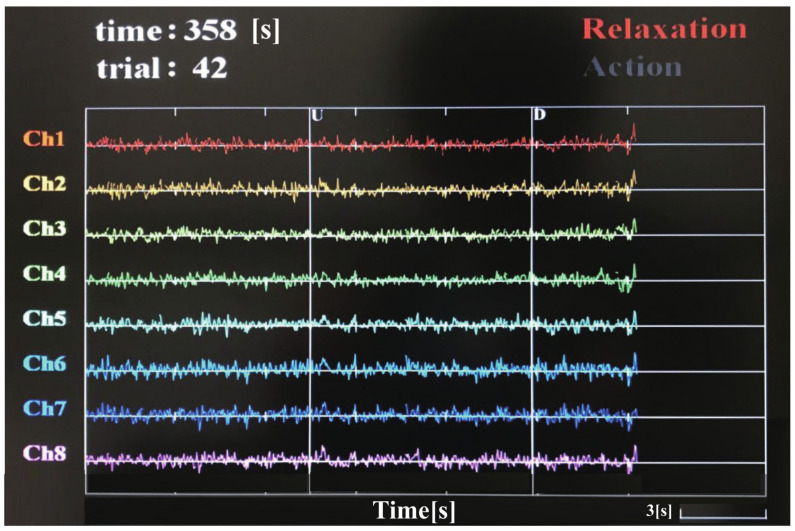
Real-time display of measured EEG signals.

The measurement environment is shown in Figure 1. The hardware system of EEG data acquisition used the products of g.tec (g.tec medical engineering GmbH, Schiedlberg, Austria). The measurement points were set at FC1, FC2, C5, C6, CP3, CP4, Cz, and Pz. The reference electrode was set to the left ear lobe, and the ground electrode was set on the mastoid of the left side. The sampling frequency of EEG signals was set to 1000 Hz. The recorded signal was displayed and checked on the screen in real-time as shown in Figure 3 to list up and discarded the trials mixed with noise such as body movement, eyes blinking, and sweating. A push-button switch was set on the table as shown in Figure 1. When the load was lifted, the button would pop up, and this moment was regarded as the onset of motion which was set as 0 s in the epoch for every trial. It was also displayed as a vertical line marked as character U (up) in the display interface shown in Figure 3. Similarly, when the load was lowered down on the table, it will display as D (down). Time in the upper left corner recorded and displayed the current time that the experiment had been carried out. Each interval of the time axis was 3 s. Trial recorded and displayed the current trial number.

#### 2.2.3. Robotic Arm for Feedback

Our developed robotic flexion and extension arm (FEA) shown in Figure 1 was used to return the angle information to the trainee in real-time during the training. This way of feedback is not only the most intuitive and easiest way for understanding, but also not prone to get bored for the trainee. This resulted in achieving better training results. FEA is a two degree of freedom robotic arm, which is assumed to be worn on the human’s arms to augment the power of the subject’s upper limb. The lengths of two link lengths are 0.3 m, which is almost the same as the lengths of an adult’s forearm and upper arm. Two Maxon 150W DC motors are used in the arm, and two harmonic drive gears are embedded in each joint, respectively. Each motor’s power is transmitted to the joint via a timing belt.

#### 2.2.4. BMI Decoder for Power Augmentation

The extended admittance control was used to design the BMI decoder to implement power augmentation [34]. The control method is described as follows.

(1) $$\tau_{h}-\tau_{0}=I\cdot \dot{\omega_{h}}+D\cdot \omega_{h}$$

where $\tau_{h}$, $\tau_{0}$, $\omega_{h}$, $\dot{\omega_{h}}$, *I* and *D* are the torque of the human, the threshold torque, the target angular velocity of FEA, the target angular acceleration of FEA, the virtual moment of inertia of the joint, and virtual damping coefficient of the joint, respectively.

The operating properties of extended admittance control are described below.

- when $\tau_{h}>\tau_{0}$, flexion mode ($\omega_{h}>0$)
- when $\tau_{h}<\tau_{0}$, extension mode ($\omega_{h}<0$)
- when $\tau_{h}=\tau_{0}$, holding mode ($\omega_{h}=0$)

With this method, the motion modes of the FEA were seamlessly switched by the values of $\tau_{h}$ and $\tau_{0}$ [35], which are generated from the power spectral density (PSD) of EEG signals described as follows.

(2) $$\tau_{h}=K\cdot PSD_{EEG}+b$$

(3) $$\tau_{0}=0.7\cdot k\cdot PSD_{EEGmax}\cdot sin\theta$$

where $PSD_{EEG}$, K,b,k, $PSD_{EEGmax}$, and θ, are PSD of EEG signals, proportionality coefficient, the EEG signals to compensate for the gravity of the subject’s arm, decrease proportionality coefficient, maximum EEG value in flexion, and the joint angle, respectively.

When the subject tries to produce a big PSD, which implies $\tau_{h}$ > $\tau_{h}$, the arm will flex. The larger the $\tau_{h}$ is, the faster the joint flexes. This is flexion mode. When the subject decreases his/her PSD to $\tau_{h}$ = $\tau_{0}$, at this instant of time, the FEA will stop the flexion nor extension. This is holding mode. If the subject wants to extend, the subject can further reduce the PSD, which leads to $\tau_{h}<\tau_{0}$. FEA will extend the joint. Moreover, the less the $\tau_{h}$ is, the faster the joint extends. This is extension mode.

### 2.3. NFB Training Procedure and Tasks

**Figure 4:**
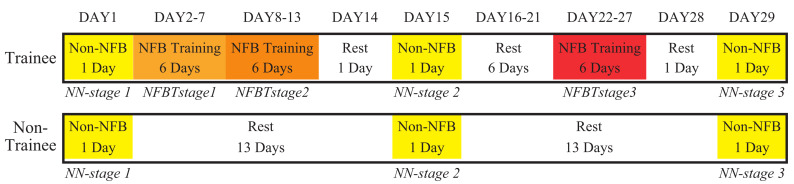
The training schedule for this study.

The training schedule is shown in Figure 4.

The trainees performed three 6-day NFB training, which is called NFBTstage1, NFBTstage2, and NFBTstage3, respectively. In NFBTstage1, the subjects were going through a period of trial and error. In this stage, various internal processes were “tried and tested” until the right mental strategies were found to produce the desired movement of the FEA. By repeating these activities during training, the respective brain mechanisms resulting EEG patterns can be stabilized. Thus, the NFBTstage2 was conducted directly behind the NFBTstage1 to further enhance and stabilize the specific changes of EEG signals. These changes were used for the NFBTstage3. The NFBTstage3 was scheduled one week after the NFBTstage2, which was designed to confirm the validity and durability of the EEG changes resulted by training. The results of these three training stages were used for the analysis of the training effect on EEG signals.

In the trainee group, a total of 720 trials of NFB training were conducted on each subject. The subject carried a box in which a 3 kg dumbbell is set in the experiment with both hands, and performed 40 trials on the shoulder joint and elbow joint in a training day, respectively. Each trial included rest, flexion movement, and extension movement. In the flexion movement, the subjects flexed their arms to an angle of about 90 degrees relative to the resting position as shown in Figure 1. The extension movement was to extend the arm from the 90-degree position back to the resting position. FEA was placed 1 m in front of the subject. During the flexion movement, the subject was required to concentrate and pay attention to the movement of the FEA, try to make the FEA flex to 90 degrees and keep the 90-degree position, and then conducted the extension movement. After the extension movement, a short break was taken to avoid fatigue. The next trial can continue at any time. Before and during the action, the subjects were instructed to avoid other movements, such as eye movements.

On the first day, the fifteenth day, and the twenty-ninth day, the experiments without NFB were conducted, which is defined as the Non-NFB task (NN task). These three experiments were labeled as NN-stage1, NN-stage2, and NN-stage3, respectively. The EEG signals’ PSD of the NN task was used to confirm whether the EEG signals changed due to NFB training by comparing the results with the control group.

### 2.4. EEG Off-Line Analysis

#### 2.4.1. EEG Preprocessing

After the EEG signals were processed by the Analog/Digital (A/D) conversion through the interface board, a 4th order Butterworth high-pass filter with a cutoff frequency of 0.1 Hz was used to eliminate the baseline drift of the recorded EEG signals. Then, the filtered EEG data of each trial were processed by ICA to remove the artifacts. Each independent component (IC)’s properties was confirmed, and the method of confirmation was as follows. Firstly, the power spectra of each IC were calculated and confirmed. The low-frequency band had higher values, and as the frequency band rose, the value gradually decreased or changed little. These ICs were regarded as EEG components. Secondly, the waveform of each trial of each IC was confirmed. In each trial, if there were the same or similar fluctuations, it indicated that this IC was related to repetitive motion tasks. On the contrary, the ICs whose power spectra were mainly concentrated on certain trials were regarded as random noise components, including body movement, head movement, etc. Moreover, in the IC topography, if the IC’s energy was concentrated in the front of the head and the low-frequency band, accompanied by the appearance of a pulse-like waveform, this IC was judged as the eye movements or blink components. After non-EEG components were removed, the epoch was extracted from the time duration 2 s before and 4 s after the onset of motion. This 6-s data was used for the EEG data analysis.

#### 2.4.2. EEG Data Analysis

This study used STFT to calculate the power spectra and the PSD. 1-s data in the rest state was used for baseline correction. 4-s data after onset was used for 512 points STFT with Hanning window. The overlap was set to 80% for updating data every 0.1 s. In this study, it is assumed that the contralateral effect of EEG signals was equal, since the motion was performed by both hands simultaneously, and to avoid the effect of left/right-handed from the subjects, the EEG signal used for NFB was from Cz.

Because of the volatility of EEG signals, it could be more effective to focus on the comparison of the EEG changes on the certain periods we care about instead of observing every sampled data. Thus, the EEG PSD was analyzed and evaluated in every 0.5 s. The results of the average value of 480 trials’ PSD of every 4 Hz in the time interval from t = −2 s to 4 s were used to clarify the specific changes in the frequency band used for NFB training. The averaged PSD in the time interval from t = 0 s to 4 s was calculated to evaluate the training effect in the different groups (trainee and non-trainee) and stages.

## 3. Results

### 3.1. Frequency Band Determination for NFB Training

**Figure 5:**
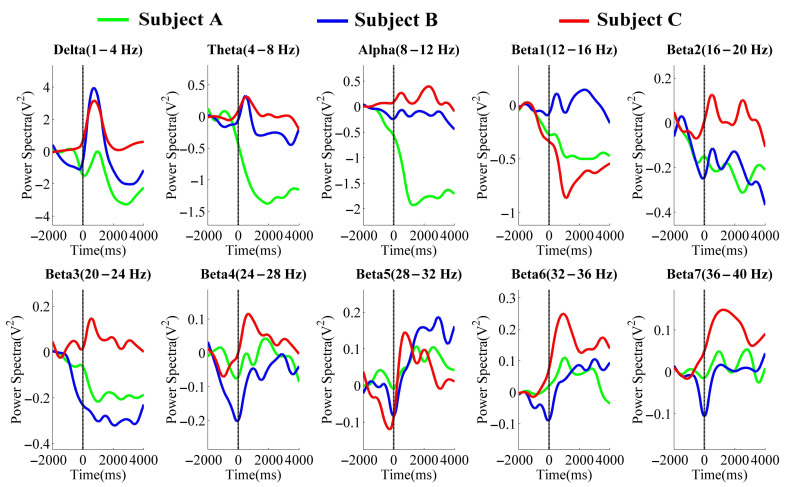
The averaged PSD values in the different frequency bands of each subject for the elbow joint.

**Figure 6:**
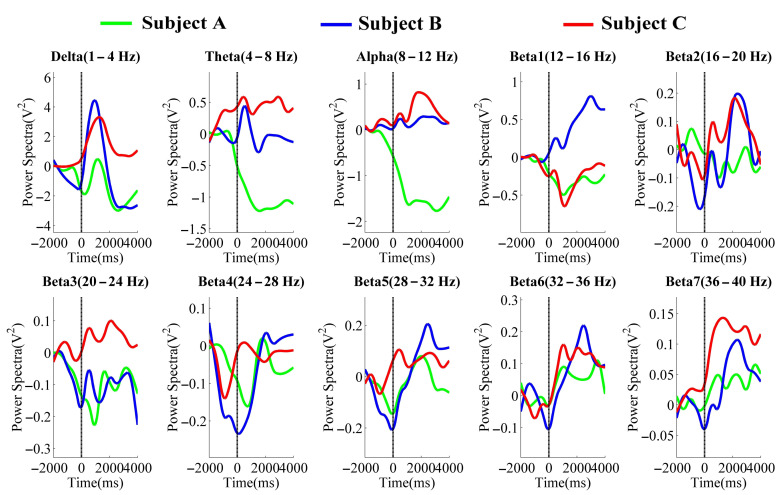
The averaged PSD values in the different frequency bands of each subject for the shoulder joint.

Figure 5 and Figure 6 were the averaged PSD results in the different frequency bands of the NFBTstage1 and NFBTstage2 of the elbow joint and the shoulder joint, respectively.

In the delta band (1–4 Hz), the changes related to the motion were observed. It started to decrease before the onset of motion and then increased significantly after the onset. The maximum value appeared around 0.5–1 s and then decayed immediately. These changes can be considered as the EEG features of the flexion movement. But after the flexion, there were no specific EEG changes of extension movement. For these reasons, the delta band is not suitable for NFB training.

Regarding the results of the theta band (4–8 Hz), changes related to the motion were also observed. But these changes were different with the different individuals. Two were increased immediately after the onset of motion, and one was attenuated significantly. From this view, the theta band is also not applicable to NFB training.

The results of the alpha band (8–12 Hz) also showed obvious individual differences. Subject A’s PSD decayed after the onset of motion, while the results of subjects B and C were increased and then without obvious changes. Thus, it is considered that EEG signals in the alpha band are affected by individual differences, and it is not easy to identify the state of rest and motion, which is also unavailable for NFB training.

In the beta band (12–40 Hz), some changes related to the motion were observed. Such as in beta4 (24–28 Hz), beta5 (28–32 Hz), beta6 (32–36 Hz), and beta7 (36–40 Hz) of the elbow joint’s results and beta5, beta6, and beta7 of the shoulder joint’s results. These changes began to attenuate before the motion and increased significantly and immediately after 0 s, which existed in all subjects’ results. The PSD of these bands also decreased during the extension movement (from 3 s after the motion started). Thus, the changes in these bands are considered as the effects of NFB training, which is also considered as the features related to the motion of the upper limbs for power augmentation. Therefore, in the NFBTstage3, the band of 28–40 Hz was used for NFB training.

### 3.2. Results of the Three NFB Training Stages

**Figure 7:**
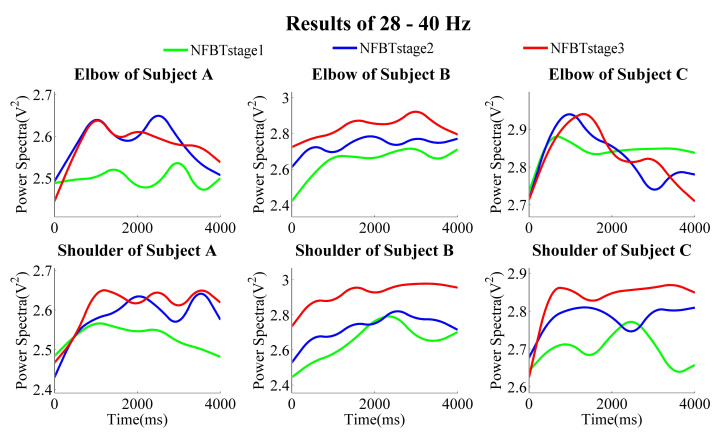
Subjects’ three NFB training stage results of averaged power spectra of 28–40 Hz during the motion of the elbow joints and the shoulder joints.

**Figure 8:**
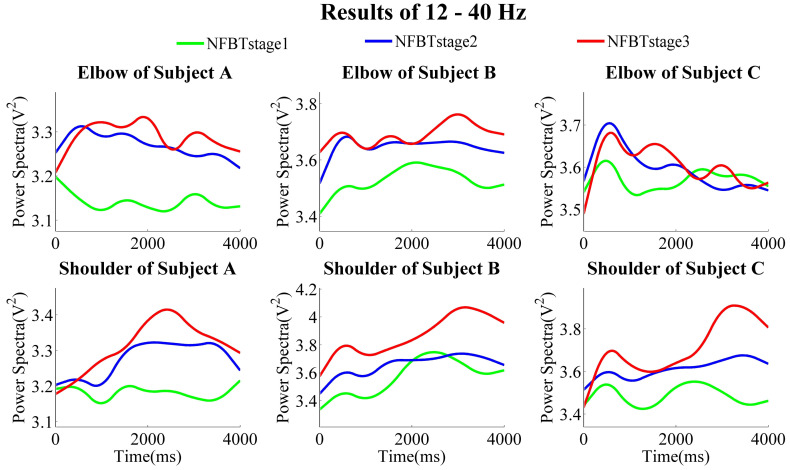
Subjects’ three NFB training stage results of averaged power spectra of 12–40 Hz during the motion of the elbow joints and the shoulder joints.

**Figure 9:**
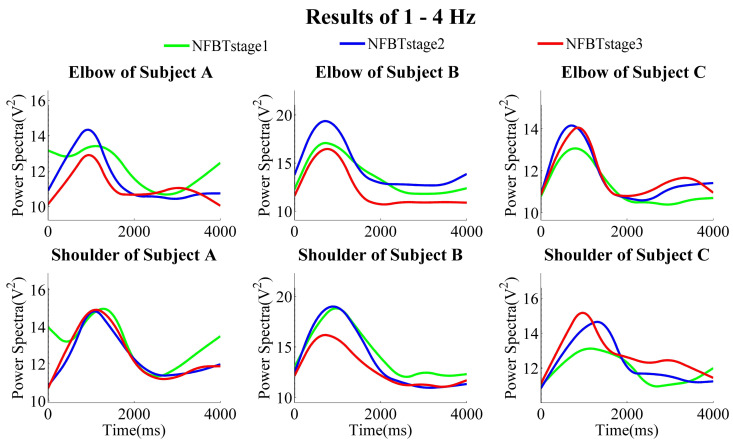
Subjects’ three NFB training stage results of averaged power spectra of 1–4 Hz during the motion of the elbow joints and the shoulder joints.

The NFB training results of each subject at the three training stages are shown in Figure 7, Figure 8 and Figure 9, which were the results of the elbow and shoulder joints in 28–40 Hz, 12–40 Hz, and 1–4 Hz, respectively.

From the results of the band of 28–40 Hz, no matter whether in the elbow joints or the shoulder joints, in general, the power was gradually enhanced from NFBTstage1 to NFBTstage3. It is considered as the effect of the training. Moreover, the training effect was not only observed in 28–40 Hz, the generally same results were also extended to the beta band of 12–40 Hz shown in Figure 8. On the other hand, there were no obvious and regular changes for both elbow and shoulder joints in the delta bands as shown in Figure 9. But the changing pattern of the delta signals was very stable, and the change characteristics became more and more obvious through the training. Thus, it is considered that the delta signals can be used for motion discrimination.

**Figure 10:**
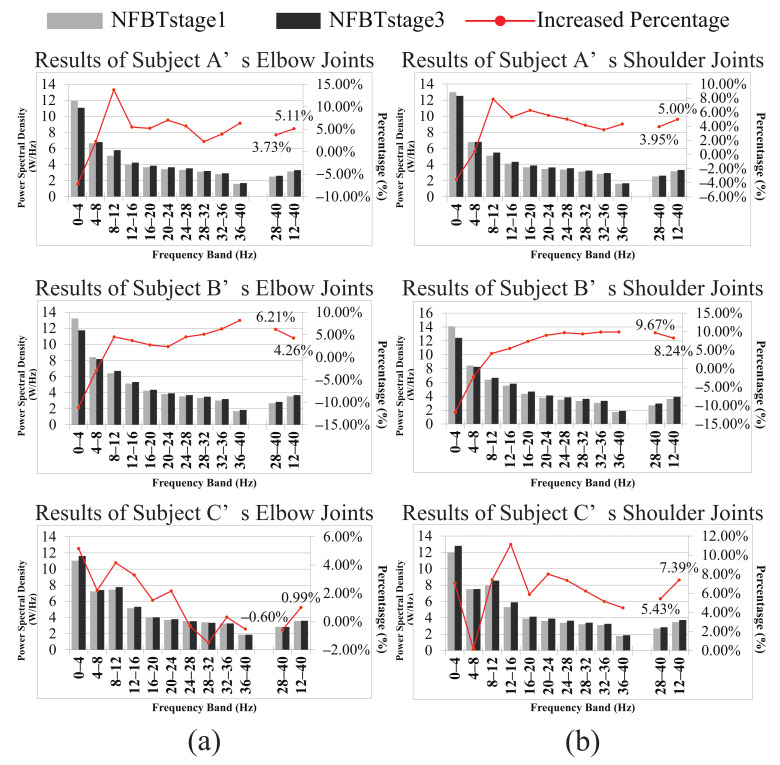
The difference and the increased percentage of each band’s PSD between NFBTstage1 and NFBTstage3 of the subject A, B, and C. (**a**) The results of the elbow joint. (**b**) The results of the shoulder joint.

Figure 10 shows the results of quantitative evaluation, including the PSD difference and the increased percentage of each band between training stages 1 and 3 of each subject. Figure (a) and (b) correspond to the results of the elbow joint and the shoulder joint, respectively. From these results, it is found that for the 28–40 Hz, compared with the NFBTstage1, the power spectra of the NFBTstage3 were enhanced by 3.12 ± 0.03% for the movement of elbow joints and 6.35 ± 0.02% for that of the shoulder joints, respectively. Moreover, this enhancement is not limited to 28–40 Hz, the power spectra of other sub-bands in the beta were also enhanced. In other words, the power spectra of the entire beta band of 12–40 Hz were also enhanced. It was enhanced by 3.45 ± 0.02% for the movement of elbow joints and by 6.88 ± 0.01% for that of the shoulder joints. Based on these results, the frequency band used for data analysis and NFB training effect evaluation between the trainee group and the control group was not only the band of 28–40 Hz, but also the frequency band of 12–40 Hz, which is used as a reference indicator for evaluation.

It can be observed from Figure 7 and Figure 8 that the results of subject C were not the same as those of subject A and subject B. The results of the shoulder joint were basically the same. The main difference was reflected in the results of the elbow joint of subject C. From the results of the elbow joint in Figure 10, it can also be found that the EEG enhancement of subject C was not as obvious as subject A and subject B. But in the NFB training experiment, subject C’s control effect of the robotic arm was the best. In the control method of the robotic arm we designed, the faster the PSD decreases, the faster the extension speed. In the same way, the faster the PSD increases, the faster the flexion speed. From Figure 7 and Figure 8, we can find that when subject C performed extension movement after the flexion, the changes in PSD were more obvious than those of subject A and subject B. But there is no such difference in them at the NFBTstage1. This also shows the effectiveness of NFB training that can enhance the EEG changes. That is to say, the EEG signals had been enhanced not only for the flexion movement but also for the extension movement. Due to the changes in Figure 8 is more obvious than that in Figure 7, it also revealed that the EEG changes existed in 12–40 Hz. It is still difficult to achieve better control of the robotic arm for the subjects without NFB training. This can be known from the results of the NFBTstage1 in Figure 7 and Figure 8, which showed the subjects were unable to achieve the extension movements well.

### 3.3. NFB Training Effect Evaluation

**Figure 11:**
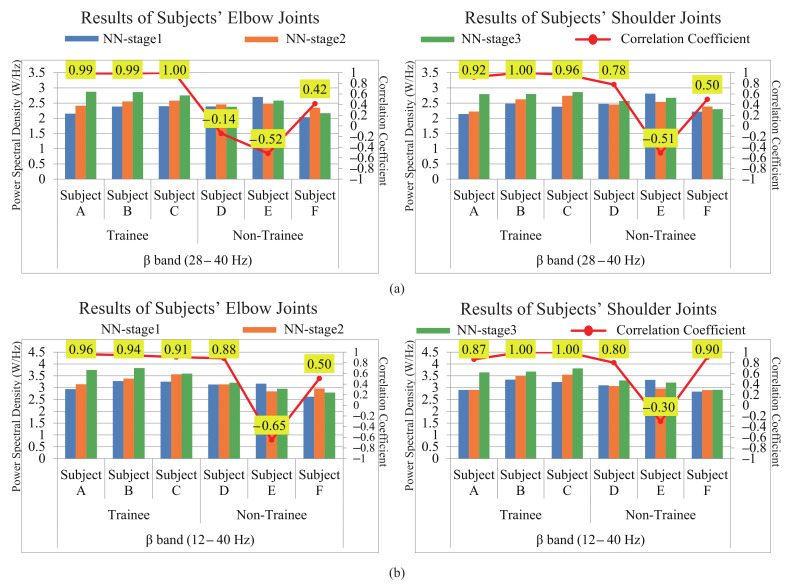
The PSD of the beta of the elbow movement and shoulder movement of the two groups. (**a**) The results of 28–40 Hz of the two groups of subjects. (**b**) The results of 12–40 Hz of the two groups of subjects.

The results of the NFB training effect evaluation are shown in Figure 11. Figure 11a,b were the results of 28–40 Hz and 12–40 Hz of the two groups of subjects, respectively. We can directly conclude from the figures that the PSD in (a) and (b) both tended to increase with the NFB training for trainee subjects A, B, and C compared to the control group. The Pearson correlation coefficient (CC) between the NFB training and the PSD of EEG signals for the elbow joints were calculated as follows.

(4) $$r=\frac{\sum_{i=1}^{n}\left(x_{i}-\bar{x}\right)\left(y_{i}-\bar{y}\right)}{\sqrt{\sum_{i=1}^{n}{\left(x_{i}-\bar{x}\right)}^{2}{\left(y_{i}-\bar{y}\right)}^{2}}}$$

where *x* and *y* were the number of training trials and the PSD of EEG signals, respectively. The same calculation of the CC for the shoulder joints was performed. The results were shown by the red markers and the CC values were drawn next to the marker. For trainees A, B, and C, the values were all above 0.87. But CC values were distributed between −0.65 and 0.88 for non-trainees D, E, and F, and the characteristics of the fluctuation were not clear. These results showed that the trainees’ PSD has a strong positive correlation with the NFB training. On the contrary, it showed that there was no obvious common change trend among the three non-trainee subjects. The reason for this difference is only considered as that all trainees were experienced NFB training while the non-trainees were not.

Moreover, a week of rest was set between the NFBTstage2 and NFBTstage3. From these results, we found that the effect of training did not disappear with the cessation of training, but was preserved. After the third stage of training, the effect was continually enhanced again. Although it is a short-term NFB training, the effect of NFB will not disappear quickly. Thus, NFB training can affect the EEG signal, and this effect will continue and be accumulated and enhanced.

Furthermore, by comparing the two results of (a) and (b) of Figure 11, we can find that the evaluation results of 28–40 Hz reflect the difference between the two groups more obviously. The CC of the trainee group’s members were all above 0.92, and that of the non-trainees did not exceed 0.78. Therefore, based on the above results, we can conclude that the EEG features for power augmentation are contained in the beta band, and most information is concentrated in the band of 28–40 Hz.

## 4. Discussion

Currently, EEG-NFB has been widely used in health care and health improvement [36,37,38,39]. But in this paper, we demonstrated a new application of the EEG-NFB approach for BMI power augmentation. The results provided some insights into the effects of NFB training and how to better adapt to BMI training, which provides an effective way for the BMI application and will promote the wide application of non-invasive BMI technology.

A functional magnetic resonance imaging(fMRI) study found that after a week of frontal-parietal beta wave NFB training, the volume of brain gray matter increased and the connectivity of white matter pathways improved [40]. Ref. [41] successfully established a new functional connection between the lateral parietal cortex and the primary motor cortex through NFB training, and found that this functional connection can be maintained for at least two months (the functional connection is referred to the synchronized change and collaborative network formed between different brain regions in the same task). Furthermore, a prefrontal beta wave NFB training study found that the beta wave changes can be maintained for at least 3 years [42]. Our study has firstly shown and verified the enhancement of the PSD of beta through the NFB training when the shoulder joints and elbow joints’ power were augmented during the flexion and extension movements. And the training effect did not immediately decline or disappear even after a week of rest, but enhanced again after the training, which shows the durability of the NFB training effect for power augmentation. It is believed that these results are of great significance for guiding the training of the BMI PA system and demonstrating the feasibility of EEG-controlled power augmentation.

For a long time, mainstream NFB solutions are based on alpha and theta waves [43,44]. It is well known that the alpha activity in EEG signals has high intra-individual variability [45]. Our results show that the results of these two bands have large individual differences, which indicates that they are not suitable for the BMI PA system.

From the results in Figure 10, it is found that the changes of the PSD after the training is inconsistent for the shoulder joints and the elbow joints. The PSD of the shoulder motion had been significantly enhanced compared with that of the elbow motion. The reasons are considered as follows. There are few movements of the shoulder joint alone in the activities of daily living. Almost all the movements such as lifting and carrying heavy objects can be achieved through the elbow joint, which leads to the fact that the movements of the elbow joint are often executed than the shoulder joint. Over many executions, the subject probably gains the ability to control the movement of the elbow without being consciously aware of how it is achieved. But for the infrequently used shoulder joint, the brain needs to provide more attention to generate the motion control commands than the elbow joint, such as to adjust the torque and to keep the balance, and so on. In other words, the brain gives priority to the elbow joint for motion execution and puts less attention on the execution process, which leads to the fact that people may perform the movements of the elbow joint subconsciously. Due to the 18-day NFB training, the brain gradually adapts to the needs of the shoulder joint movements. Therefore, the PSD enhancement of the shoulder motion is more significant than that of the elbow joint, and the frequency bands that are enhanced by the motion of the shoulder joints are also the features of the elbow joints.

It is known that long-term training can better overcome the difficulties in learning to control the non-invasive BMI. But the results of this study show that only a short period of NFB training can effectively improve the subjects’ ability to use BMI to achieve power augmentation. This indicates that NFB training can enhance human mind control. It also provides a new idea and method for the EEG feature extraction and the BMI decoder design.

If the decoder is trained during passive observation, brain control was superior without arm movement [46]. Similarly, if the decoder is trained during movement, brain control was better when the arm was moving [47]. In other words, the performance will be better if the training and testing conditions are congruent. The differences may be related to whether there is somatosensory feedback. Thus, it is necessary to discuss the effect of somatosensory when putting on the exoskeleton for power augmentation in the next step. Another limitation of our research is the small number of subjects. However, the purpose of this study was to demonstrate the feasibility of NFB training for a BMI system construction for the power augmentation, to clarify the specific changes in the EEG signals, to verify the effectiveness of the training. As a result, the results of these six subjects were sufficient to demonstrate the feasibility and effectiveness.

## 5. Conclusions

The goal of this pilot study was to clarify and evaluate the specific EEG changes when controlling a power augmentation device to follow the human movement in healthy people. Therefore, an EEG-NFB BMI power augmentation system was constructed. We used an actual robotic arm for feedback, not limited to the abstract visual feedback displayed on a two-dimensional plane. The NFB training was used to enhance and clarify the specific changes in EEG signals during flexion and extension of the elbow and shoulder joints. Three NFB training stages were designed, and each stage lasted for 6 days. Then we used STFT to confirm and evaluate the changes in the time-frequency domain. We extracted and confirmed the PSD of each frequency band in the 0 to 40 Hz frequency band. The results showed that the EEG beta (12–40 Hz) PSD increased after the NFB training for the movement of the elbow and the shoulder joints. Moreover, EEG beta PSD showed sustained enhancement during the 3-stage training, which revealed that even the short-term training could enhance EEG signals significantly. Furthermore, the training effect of the shoulder joints was more obvious than that of the elbow joints. Since the functional areas of the shoulder and elbow joints in the brain are very close, currently there is no good way to distinguish them through EEG signals. Our results provide a potential way to distinguish the movement of the shoulder and elbow joints.

This BMI power augmentation system demonstrates a new application of a well-known and widely used NFB method in power augmentation. Our finding suggested that healthy people could benefit from NFB training for BMI power augmentation. It also provides a basis for the feature extraction and BMI decoder construction in the next step. Our results may even provide insights into how the neural effects of NFB can be better applied to the BMI power augmentation system, enhance the human brain control ability, and promotes the wide application of non-invasive BMI technology.

## Data Availability

The data presented in this study are available on request from the corresponding author. The data are not publicly available due to ethics and privacy.

## Abbreviations

The following abbreviations are used in this manuscript:

BMI	brain-machine interface
EEG	electroencephalography
PA	power augmentation
EMG	electromyography
NFB	neurofeedback
NFBT	neurofeedback training
FFT	fast Fourier transform
ICA	independent component analysis
PCA	principal component analysis
TFD	time-frequency distributions
SNR	signal-to-noise ratio
SMR	sensorimotor rhythm
RTAI	real-time application interface
FEA	flexion and extension arm
PSD	power spectral density
A/D	analog/digital
IC	independent component
STFT	short-time Fourier transform
CC	correlation coefficient
fMRI	functional magnetic resonance imaging
